# Comprehensive Evaluation of Varietal Differences in Glutinous Rice: Multidimensional Assessment of Cooking Quality and Processing Characteristics

**DOI:** 10.3390/foods15071215

**Published:** 2026-04-02

**Authors:** Qianqian Zhang, Bangdi Liu, Min Zhang, Lina Guan, Guodong Ye, Min Zhang, Jing Sun, Sixuan Li

**Affiliations:** 1 School of Food and Health, Beijing Technology and Business University, Beijing 100048, China; zhangzq09@163.com (Q.Z.); 18238370072@163.com (L.G.); lvzhuojia@163.com (S.L.); 2 Ministry of Agriculture and Rural Affairs, Academy of Agricultural Planning and Engineering, Beijing 100125, China; ziyanc2023@163.com (B.L.); cynthiasj@163.com (J.S.)

**Keywords:** glutinous rice, cooking quality, flavor profile, processing suitability

## Abstract

Varietal characteristics significantly influence the quality of glutinous rice and its products; however, comprehensive studies on its cooking and processing properties remain limited. This research systematically evaluated the impact of varietal differences on these qualities, providing guidance for the industrial production and processing of glutinous rice. Seven widely cultivated varieties were selected to evaluate the physicochemical properties, processing performance, and eating quality. Key findings reveal that *WK* (*Wanken*) excels in water-binding capacity, exhibits a distinct protein composition, and possesses a compact texture. *ZK* (*Zhongke*) demonstrates superior thermodynamic properties, exceptional gel consistency, and strong anti-retrogradation ability, making it ideal for instant food processing. *WL* (*Wuliang*) achieves peak viscosity and high thermal stability, while *ZZ* (*Zhennuo*) exhibits optimal flavor quality. The innovation of this study lies in elucidating the functional relationships between multi-scale parameters—such as water mobility, protein fraction distribution, thermal properties, and volatile flavor compounds—and specific quality traits, thereby providing a theoretical basis for precision breeding and process-cultivar alignment.

## 1. Introduction

Glutinous rice (*Oryza sativa* L. var. *glutinosa*), as a staple grain with unique edible and functional properties, holds a pivotal position in Asian culinary culture and the food industry [1]. Unlike nonglutinous rice, glutinous rice contains almost no amylose in its endosperm, with its starch primarily composed of amylopectin. This results in its signature sticky, soft, and glutinous texture after steaming or boiling [2]. This unique physicochemical property makes it an indispensable ingredient in numerous traditional and modern food industries, such as the well-known zongzi (rice dumplings), nian gao (rice cakes), tangyuan (glutinous rice balls), glutinous rice pastries, and alcoholic beverages [3]. However, a core issue that has long plagued industrial development is the significant variation in texture, flavor, and processing stability among different glutinous rice varieties. This unpredictability in raw material quality severely hampers standardized production and the high-end development of products.

While varietal importance is well-recognized, research on glutinous rice faces clear limitations. Most studies analyze the basic physicochemical properties of only a few cultivars, resulting in a narrow scope focused on single varieties or specific products [4]. Even where studies involving multiple glutinous rice cultivars do exist, they have largely been confined to fundamental chemical and pasting properties, underscoring the necessity of establishing a broader, multi-dimensional research framework [5]. A disconnect also exists between microstructural properties and macroscopic functionality. Although thermal behaviors (e.g., GT, ΔH via DSC) and pasting properties (e.g., RVA) are well-documented, the data often remain descriptive [6]. Bridging this gap requires integrating parameters like water mobility (LF-NMR), protein content, and thermal-rheological data [7]. Similarly, cooking quality assessment remains fragmented, typically relying on instrumental texture (TPA) or subjective sensory evaluation with inadequate correlation between them [5]. While early studies linked some sensory attributes to physicochemical parameters, and recent E-nose/GC-MS analyses have connected volatiles to sensory profiles in products like huangjiu, a holistic approach integrating color, moisture, texture, and flavor data is still lacking [8].

Therefore, to address this research gap, this study systematically compared and analyzed the physicochemical properties (water mobility via LF-NMR, protein content via Kjeldahl, and protein fractions via Osborne fractionation), processing characteristics (thermal properties via DSC and pasting properties via RVA), and cooking quality (texture profile, color, volatile flavor compounds via GC-MS, and sensory evaluation) of seven widely cultivated glutinous rice varieties from major Chinese production regions. By integrating these multi-scale parameters, this study aims to elucidate the functional mechanisms by which varietal differences influence quality formation, providing a scientific basis for precision processing and cultivar-specific industrial applications.

## 2. Materials and Methods

### 2.1. Materials and Chemicals

Seven glutinous rice cultivars were obtained from the Chinese Academy of Agricultural Sciences. These cultivars were selected based on their diverse geographical origins, significant cultivation volumes, and known end-use applications to ensure a representative sample of the Chinese glutinous rice market. Jietiandao (JT) from Anhui Province represents a widely adapted landrace. Wanken No. 5 (WK) and Zhongkenuo No. 5 (ZK) from Anhui Province are the varieties with the highest cultivation and processing volumes in Huaiyuan, a major production hub. Longjing (LJ) from Heilongjiang Province is the predominant glutinous rice cultivar in Northeast China, demonstrating exceptional yield and cultivation area. Tenuo (XY) from Henan Province is a principal commercial variety in a high-yielding region. Wuliang (WL) from Sichuan Province is a specialized cultivar optimized for alcoholic fermentation, while Zhenzhudao (ZZ) from Hubei Province exhibits favorable comprehensive quality parameters among the domestic varieties. All cultivars were cultivated during the 2024 cropping season under identical agronomic practices. After harvest, the paddy grains were stored at 4 °C to ensure sample stability.

Rice preparation: Paddy grains were dehusked using a laboratory husker (JLG-II, Zhejiang Top Cloud Agri Technology Co., Ltd., Hangzhou, China), then milled with a rice polisher (JNM-III, Zhejiang Top Cloud Agri Technology Co., Ltd., Hangzhou, China) under controlled parameters (20% grinding rate) to achieve first-grade processing standards. The processed rice was vacuum-sealed and stored at −20 °C.

Rice flour preparation: Milled rice was ground through an 80-mesh sieve (180 μm) using a cyclone mill (FW-100, Beijing Creta Technology Co., Ltd., Tianjin, China), then vacuum-packed and stored at −20 °C.

Cooked rice preparation: 20 g of rice was weighed into a lidded aluminum container, rinsed three times with distilled water, drained, and hydrated with distilled water at a 1.3:1 water-to-rice ratio. After 30 min of room temperature soaking, samples were steamed for 30 min, followed by 10 min equilibration in an electric rice cooker (MB-FZ4086, Midea Group Co., Ltd., Beijing, China).

All chemical reagents were obtained from GenBase Biotechnology Co., Ltd. (Beijing, China).

### 2.2. Determination of Physicochemical Indicators

#### 2.2.1. Water Mobility and Distribution

Moisture proton relaxation times were determined using a low-field NMR analyzer (Niumag, Shanghai, China) with Carr–Purcell–Meiboom–Gill (CPMG) sequences according to [9]. Approximately 2.0 g of glutinous rice was equilibrated in NMR tubes at ambient temperature (25 °C) for 30 min prior to measurement. Transverse relaxation time (T2) was recorded with the following parameters: 90° hard pulse width (P1) = 17.52 μs; 180° hard pulse width (P2) = 34.48 μs; relaxation delay (TW) = 1000 ms; sampling points (TD) = 40,028; echo number (NECH) = 500; echo time (TE) = 1.0 ms; receiver gain (PRG) = 2; radiofrequency delay (RFD) = 0.08 ms; scan repetitions (NS) = 4.

#### 2.2.2. Determination of Protein Contents

The protein content of glutinous rice was determined by the Kjeldahl method, whereby the crude protein content was calculated by measuring the nitrogen content of the sample and multiplying it by a conversion factor of 6.25 [10].

#### 2.2.3. Determination of Graded Protein

Following the Osborne fractionation procedure with minor modifications [11], 4 g of glutinous rice flour from each cultivar was sequentially extracted with distilled water, sodium chloride (NaCl), ethanol, and sodium hydroxide (NaOH) to isolate the albumin, globulin, prolamin, and glutelin fractions, respectively. Protein extraction efficiency was monitored throughout the process. Protein concentrations in the extracts were quantified using a microplate reader (Multiskan GO, Thermo Scientific, Waltham, MA, USA) by measuring absorbance at 562 nm against a bovine serum albumin (BSA) standard curve.

#### 2.2.4. Determination of Gel Consistency

According to GB/T 22294-2008 [12], the gel consistency of glutinous rice flour was determined. A sample (0.1000 g, passed through 100-mesh sieve) was placed in a test tube, mixed with 0.2 mL thymol blue indicator and 2.0 mL 0.200 mol/L KOH solution, then heated in a boiling water bath for 8 min. After cooling at room temperature for 5 min, the tube was horizontally placed in an ice-water bath (0 °C) for 20 min, then horizontally laid at 25 ± 2 °C for 1 h. The gel length (mm) was measured to evaluate the gel consistency.

#### 2.2.5. Determination of Alkali Elimination Value

Alkali elimination value was determined according to NY/T 83-2017 [13]. Six whole milled rice grains were soaked in 10 mL of 1.7% KOH solution at 30 °C for 23 h, and the degree of disintegration was visually scored on a 7-point scale.

### 2.3. Determination of Processing Quality

#### 2.3.1. Thermodynamic Properties

Methods of referencing in [14] were employed to analyze the thermodynamic characteristics using a differential scanning calorimeter (DSC 250, TA Instruments, New Castle, DE, USA). Approximately 3.5–4.0 mg of glutinous rice flour was weighed into an aluminum crucible, mixed with double its mass of distilled water, hermetically sealed, and equilibrated at 4 °C overnight. Samples were heated from 30 °C to 120 °C at a rate of 5 °C/min under nitrogen purge (50 mL/min). An empty crucible served as the reference. Gelatinization parameters, including onset temperature (To), peak temperature (Tp), conclusion temperature (Tc), and enthalpy (ΔH), were derived from the thermograms.

#### 2.3.2. Pasting Properties

Pasting characteristics were analyzed using a Rapid Visco Analyzer (RVA, PVA-5280, Newport Scientific, Jessup, MD, USA). Methods of referencing in [15] were used. Approximately 3.0 g of glutinous rice flour was mixed with 25 mL of distilled water in an RVA test canister. The temperature profile comprised: holding at 50 °C for 1 min, heating to 95 °C over 4 min, maintaining at 95 °C for 2.5 min, cooling to 50 °C within 4 min, and holding at 50 °C for 1.5 min.

### 2.4. Determination of Cooking Edible Quality

#### 2.4.1. Texture Profiles

Texture properties were evaluated using a texture analyzer (TA.XT Plus, Stable Micro Systems, Godalming, UK) following [16] with modifications. Precisely 8 g of cooked rice was molded into cylindrical discs (32 mm diameter × 8 mm height) using a compression fixture. A P36/R probe was employed in texture profile analysis (TPA) mode with the following parameters: pre-test speed 1.0 mm/s, test speed 0.5 mm/s, post-test speed 1.0 mm/s, trigger force 5 g, and 50% strain. Ten independent replicates were performed per sample.

#### 2.4.2. Color of Glutinous Rice

Color parameters of cooked glutinous rice from different cultivars were measured using a chroma meter (CR-400, Konica Minolta, Tokyo, Japan). Samples were evenly distributed in Petri dishes, and (L*), redness (a*), and yellowness (b*) were recorded. Calibration was made via a white tile (Y = 81.9, x = 0.3222, c = 0.3401) before testing. Afterward, the whiteness (W) was calculated based on Formula (1) [17]:

(1) $$W\;=\;100\;-\sqrt{{\left(100\;-\;L^{*}\right)}^{2}+{a^{*}}^{2}+{b^{*}}^{2}}$$

#### 2.4.3. Flavor Profile Analysis

##### Electronic Nose (E-Nose)

The flavor profiles of different cooked rice samples were measured according to the method by [18]. Freshly cooked rice samples (10.0 g) were placed in 50 mL sampling vials and immediately sealed with Parafilm to minimize volatile loss. After equilibration at 80 °C for 15 min in a temperature-controlled chamber, headspace analysis was conducted using an electronic nose system (FOX-4000, Alpha MOS). The sampling needle was automatically inserted into the vial headspace, and volatiles were delivered to the sensor array at a constant flow rate of 300 mL/min. Operational parameters included: analysis duration (60 s), sample preparation interval (5 s), and sensor purge time (100 s). Triplicate measurements were performed for each sample.

##### Gas Chromatography-Mass Spectrometry (GC-MS)

Volatile compounds in cooked glutinous rice were extracted via solid-phase microextraction (SPME). Triplicate measurements were performed for each sample. Precisely 5.0 g of cooked rice was transferred to a 20 mL headspace vial, spiked with 1 μL of 2-methyl-3-heptanone (0.816 μg/μL, internal standard), and incubated at 80 °C for 15 min under agitation. SPME fiber (50/30 μm DVB/CAR/PDMS, Supelco, Darmstadt, Germany) was exposed to the headspace for 40 min of adsorption, followed by 5 min of desorption in the GC-MS injection port.

GC conditions: DB-WAX capillary column (30 m × 0.25 mm × 0.25 μm; Agilent, Santa Clara, CA, USA); splitless injection mode; injector temperature 250 °C; helium carrier gas (99.99% purity) at 1.2 mL/min. Temperature program: initial hold at 40 °C for 3 min, ramp to 200 °C at 5 °C/min (hold 35 min), then increase to 230 °C at 10 °C/min (hold 3 min); total run time: 41 min. MS conditions: electron ionization (EI) at 70 eV; transfer line 280 °C; ion source 230 °C; quadrupole 150 °C; mass range 55–500 *m*/*z* [18].

Volatile compounds were identified by matching the mass spectra and retention indices (RIs) against the NIST 11.0 database (https://webbook.nist.gov, accessed on 16 April 2025) using n-alkanes (C7–C26) as RI calibration standards. Compounds with spectral match factors > 80% were considered valid. Quantification was performed using 2-methyl-3-heptanone as an internal standard, with volatile concentrations calculated via Formula (2):

(2) $$C\;(\mu g/\mathrm{kg})=\frac{P_{C}}{P_{\mathrm{is}}}\;\times \;C_{\mathrm{is}}\;\times \;1000/m_{0}$$

where C is the volatile compound concentration (μg/kg), C_is_ is the internal standard concentration (μg/μL), P_c_ is the volatile compound peak area, P_is_ is the internal standard peak area, and m_0_ is the sample mass (g).

#### 2.4.4. Sensory Evaluation of Cooked Glutinous Rice

The sensory acceptability of cooked rice samples was evaluated by a trained panel (*n* = 10, 5 females/5 males, aged 20–30 years) following the methodology described by Mi et al. [18].

All panelists were students and researchers from the School of Food and Health, Beijing Technology and Business University, with prior experience in the sensory evaluation of cereal products. They underwent two additional training sessions specific to glutinous rice sensory attributes (odor, appearance, palatability, stickiness, and texture of cold rice) using a structured scoring system stipulated by the GB/T 15682-2008 standard [19] (detailed criteria are provided in Table A1).

Ethical Statement and Informed Consent: According to the institutional policies of Beijing Technology and Business University and relevant national guidelines, this category of low-risk sensory analysis involving the tasting of common food products does not require formal approval from an Institutional Review Board (IRB). This study was conducted in accordance with the ethical principles for sensory science. All panelists were fully informed about the study procedures and provided their written informed consent prior to their participation. They were also advised of their right to withdraw from the session at any time without penalty with an institutional statement confirming this ethical compliance.

To ensure reproducibility, the evaluation was conducted in triplicate on separate days, and the scores were analyzed for consistency. The results presented are the mean of the triplicate evaluations.

### 2.5. Statistical Analysis

All experiments were performed in a completely randomized design with at least three independent replicates (*n* = 3 or *n* = 6). Data were processed using Microsoft Excel 2022 (Microsoft Corp., Redmond, WA, USA) and analyzed through one-way analysis of variance (ANOVA) with Duncan’s multiple comparison test (*p* < 0.05) to determine significant differences among cultivars. All statistical analyses were performed using IBM SPSS Statistics 20.0 (IBM Corp., Chicago, IL, USA). Pearson correlation coefficients were calculated to assess the relationships among quality parameters. Graphical representations were generated using Origin 2022 (OriginLab Corp., Northampton, MA, USA).

## 3. Results

### 3.1. Analysis of Physicochemical Indicators

#### 3.1.1. Analysis of Water Distribution

**Figure 1:**
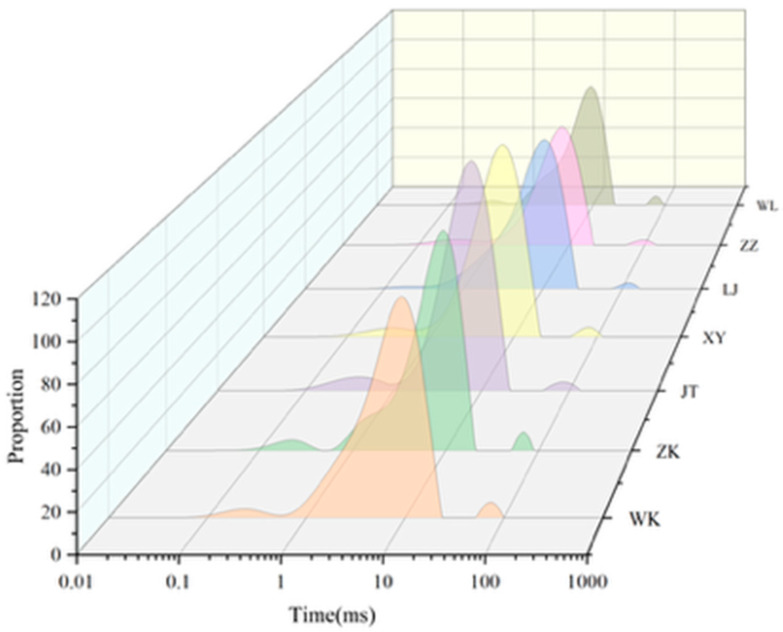
Moisture distribution patterns of different glutinous rice cultivars. The T_2_ relaxation spectra show the distribution of bound water (T_21_), immobilized water (T_22_), and free water (T_23_). Distinct differences in peak intensities reflect varietal variations in water-binding capacity.

Transverse relaxation times (T_2_) reflect water mobility, where *T*_21_, *T*_22_, and T_23_ correspond to bound water, immobilized water, and free water, respectively. The relative peak areas (*A*_21_, *A*_22_, *A*_23_) represent the population and spatial distribution of water protons in distinct domains [20]. As shown in Table 1, JT and ZZ exhibited significantly higher A_21_ values than other cultivars (*p* < 0.05), indicating enhanced water-binding stability through stronger hydrogen bonding or denser starch networks [21]. In contrast, WK, ZK, XY, LJ, and WL showed lower A_21_ values with minimal inter-varietal differences. *T*_22_ showed no significant differences among cultivars, likely attributed to the uniformly high amylose content in glutinous rice that governs similar proportions of surface-bound water. For *T*_23_ distribution, LJ demonstrated the lowest A_23_ value (*p* < 0.05), suggesting exceptionally low free water content and reduced water activity, critical for inhibiting microbial proliferation and chemical deterioration during storage. Conversely, ZK and WL exhibited elevated *A*_23_ values, implying greater free water mobility, potentially associated with loose amylopectin architectures [3]. Notably, ZK maintained the highest *A*_22_ despite moderate *A*_21_, implying a possible compensatory effect of the gel network through optimized starch–water interactions. The waterfall plot (Figure 1) revealed distinct hydration architectures: JT displayed a “high-bound, low-free” compact structure, whereas ZK showed a “low-bound, high-free” porous configuration, with ZK’s free water proportion exceeding JT’s by 26.6%. This moisture distribution pattern critically influences cooking quality—elevated bound water enhances textural stability, while excessive free water may accelerate sensory deterioration.

#### 3.1.2. Analysis of Protein Content

Protein content serves as a critical nutritional quality indicator for glutinous rice, directly determining the commercial value and nutritional profile of derived food products. As shown in Table 2, the total protein content exhibited significant varietal differences (*p* < 0.05). WK showed the highest protein content (8.37%), significantly exceeding other cultivars. Its minimal standard deviation further indicated superior content stability—a critical trait for industrial applications, likely conferring enhanced functional performance (e.g., gelation capacity, textural retention) [22]. ZK (7.98%) and JT (7.75%) ranked second and third, respectively, but both differed significantly from WK. XY (7.44%) and LJ (7.08%) displayed progressively lower values, while ZZ (6.46%) and WL (6.51%) showed the weakest performance. Their low-protein characteristics may restrict competitiveness in high-protein food formulations. Despite comparable means, WL demonstrated a lower coefficient of variation than ZZ, suggesting superior content stability. Collectively, WK’s high protein content and exceptional stability position it as a premium functional ingredient.

Further fractional protein extraction analysis revealed distinct protein distribution characteristics among the glutinous rice cultivars. Sequential isolation of albumin, globulin, prolamin, and glutelin fractions demonstrated significant inter-varietal differences (*p* < 0.05). The albumin content was highest in JT (16.98%), while ZK contained the highest proportion of glutelin (62.3%). In contrast, WK was characterized by a relatively high globulin content (11.51%) compared to other varieties. These differential distributions are functionally significant: the enriched hydrophilic albumin in JT and globulin in WK may enhance product palatability through improved solubility and emulsifying capacity, facilitating hydration and texture softening [23]. Conversely, ZK displayed elevated prolamin (9.8%) and glutelin (62.3%) levels. Conversely, the elevated levels of hydrophobic prolamin and glutelin in ZK likely contribute to structural integrity in processed foods due to their superior thermostability and acid resistance, which is advantageous for thermally processed or acidic formulations [24].

#### 3.1.3. Analysis of Gel Consistency and Alkali Elimination Value

The results of the gel consistency and alkali elimination value measurements are shown in Table 3. Statistical analysis showed that there were significant differences (*p* < 0.05) in the gel consistency and alkali elimination value of different glutinous rice varieties. Gel consistency, which reflects the softness and adhesiveness of cooked rice, further supports the textural characteristics observed in sensory evaluation. Gel consistency ranged from 88 mm (WL) to 116 mm (ZK), while alkaline elimination values exhibited broad variability (2.8–6.6 grade), with ZK showing the highest value and XY the lowest. Cultivars with high gel consistency (e.g., ZK) are preferable for processing applications requiring viscous stability [25]. Notably, the independent variation between gel consistency and alkaline elimination traits underscores the necessity of trait-specific optimization for targeted industrial applications.

Alkaline elimination values further revealed starch solubility differences among cultivars. ZK and JT displayed superior alkaline elimination values, indicative of enhanced starch solubility under alkaline conditions and reduced risks of incomplete gelatinization during cooking. The highest alkaline elimination value in ZK suggests that its starch molecules are more susceptible to hydrolysis in alkaline media, yielding softer texture and improved digestibility [26]. Overall cooking performance analysis positioned ZK as producing exceptionally soft cooked rice, while WK exhibited intermediate textural properties with favorable cooking efficiency based on these indices.

### 3.2. Analysis of Processing Quality

#### 3.2.1. Analysis of Thermodynamic Properties

Thermodynamic analysis of seven glutinous rice cultivars (Table 4) revealed significant varietal differences (*p* < 0.05) in gelatinization parameters: onset temperature (T_o_), peak temperature (T_p_), conclusion temperature (T_c_), and enthalpy change (ΔH). The WL cultivar exhibited superior thermal stability, with significantly higher T_o_ (69.62 °C), T_p_ (76.55 °C), T_c_ (84.17 °C), and ΔH (14.15 J/g) compared to other varieties, suggesting higher structural order or crystalline stability within the starch granules, which requires greater energy input for gelatinization [27]—a characteristic advantageous for high-temperature industrial processing. In contrast, ZK and JT demonstrated markedly lower T_o_ (62.08 °C, 62.68 °C) and T_p_ (68.10 °C, 67.41 °C), indicative of low-temperature gelatinization initiation likely associated with reduced amylopectin content or loose molecular packing. Notably, XY showed elevated T_o_ (67.83 °C), while ZZ displayed high T_c_ (78.58 °C), though their respective ΔH values diverged significantly (9.68 J/g vs. 12.38 J/g). This disparity implies differential energy requirements despite comparable phase stability at specific temperature intervals, potentially attributable to variations in starch branching patterns or granular morphology [28]. The ΔH dichotomy between WL (14.15 J/g) and JT (8.72 J/g) highlights structural contrasts: WL’s high crystallinity versus JT’s amorphous dominance. These thermodynamics dictate processing suitability—WL’s robust thermal resistance aligns with prolonged high-temperature processing, while ZK/JT’s rapid gelatinization kinetics favor time-sensitive applications requiring swift structural modification.

#### 3.2.2. Analysis of Pasting Properties

The study of the pasting characteristics of the seven glutinous rice samples in Table 5 revealed significant differences (*p* < 0.05) in the thermal stability of the different varieties. Peak viscosity (PV) and final viscosity (FV), critical indicators of starch swelling capacity, demonstrated that WL and ZZ possessed superior water absorption and granule expansion properties, likely attributable to high-molecular-weight amylopectin or elevated crystalline domain proportions. Notably, WL exhibited the highest breakdown viscosity (BV), indicating pronounced viscosity reduction under high-temperature shear conditions, which suggests structural vulnerability potentially arising from loosely packed amylopectin long chains or insufficient hydrogen-bond stabilization [29].

In contrast, WK and ZK displayed lower BV values combined with moderate gelatinization temperatures (GT), signifying enhanced structural integrity during thermal processing. This stability may derive from dense granule surface architectures or synergistic interactions between amylose and k-mamylopectin [30]. Setback viscosity (SV) variations further reflected starch retrogradation potential. Elevated SV values in WL and ZZ implied strong recrystallization propensity post-cooling, making them suitable for frozen glutinous products requiring long-term textural retention. However, XY’s high SV exhibited substantial deviation in FV and SV metrics, suggesting molecular heterogeneity in starch chain alignment that may increase the sensitivity to processing variables (e.g., moisture, temperature), potentially compromising product consistency.

JT’s low SV and minimal GT (67.8 °C) highlighted its suitability for rapid gelatinization processes with low retrogradation demands, such as instant rice products. GT variations further elucidated thermal responsiveness diversity: WL’s maximal GT (78.2 °C) aligned with its high-viscosity profile, likely attributable to the superior thermal stability of crystalline lamellae within starch granules [31]. Conversely, ZK and JT’s reduced GT values (71.3 °C, 69.6 °C) suggested higher amorphous region ratios or weaker intermolecular forces, favoring energy-efficient low-temperature processing.

### 3.3. Analysis of Steaming Edible Quality

#### 3.3.1. Analysis of Texture Properties

Hardness, springiness, chewiness, adhesiveness, cohesiveness, and resilience were selected as primary indicators to evaluate cooked glutinous rice texture (Table 6). Significant varietal differences in textural parameters revealed distinct processing adaptability among the cultivars. The WK and ZK groups exhibited superior hardness and gumminess, suggesting a denser protein network or stable cross-linking system formed during processing, potentially attributed to genetic variations or microstructure reinforcement through steaming protocols [32]. Conversely, JT and XY displayed significantly lower hardness and chewiness, likely associated with enhanced water-binding capacity or looser matrix alignment, making them suitable for applications requiring delicate textures. LJ and ZZ showed intermediate elasticity and cohesiveness, indicative of balanced textural properties possibly linked to moderate matrix binding or homogeneous component distribution. Notably, WL demonstrated exceptional resilience but overall weak textural strength, which specific processing conditions may disproportionately influence.

Comprehensive analysis identified significant differences in all textural parameters across cultivars. WK and ZK exhibited optimal textural performance: their hardness surpassed other samples significantly, while springiness, chewiness, and adhesiveness reached peak values, reflecting compact and resilient textures correlated with dense starch gel networks [33]. Enhanced cohesiveness and resilience further confirmed the structural stability and recovery capacity.

In contrast, JT and XY showed minimal textural strength, with hardness, chewiness, and adhesiveness significantly lower than WK/ZK, combined with poor cohesiveness and resilience. These characteristics suggest loosely organized matrices potentially linked to incomplete gelatinization or heterogeneous moisture distribution. LJ and ZZ displayed moderate hardness but reduced adhesiveness, indicating weak intergranular bonding. WL’s textural paradox—high resilience with low hardness and chewiness—implies processing parameter sensitivity or inherent material limitations.

#### 3.3.2. Analysis of Color

Statistical analysis of colorimetric parameters (L*, a*, b*) and whiteness (W) of the seven glutinous rice varieties showed significant varietal differences (*p* < 0.05) in color characteristics, as shown in Table 7. WK and ZK exhibited the highest lightness, indicating superior light reflectance likely associated with elevated starch crystallinity or compact granule alignment. Conversely, JT and WL showed minimal L* values, suggesting structural porosity or light-absorbing components (e.g., phenolic compounds). All cultivars displayed greenish hues (negative a* values), with WL demonstrating the most pronounced green bias (a*), potentially linked to chlorophyll precursor retention or differential oxidative states during processing. ZZ exhibited the highest yellowness, likely attributable to carotenoid accumulation or Maillard reaction products, whereas ZK showed near-neutral b* [34]. W trends paralleled L* values, with WK and ZK achieving superior whiteness, consistent with the optical regulation by starch surface smoothness.

#### 3.3.3. Flavor Characteristics

##### Electronic Nose Analysis

**Figure 2:**
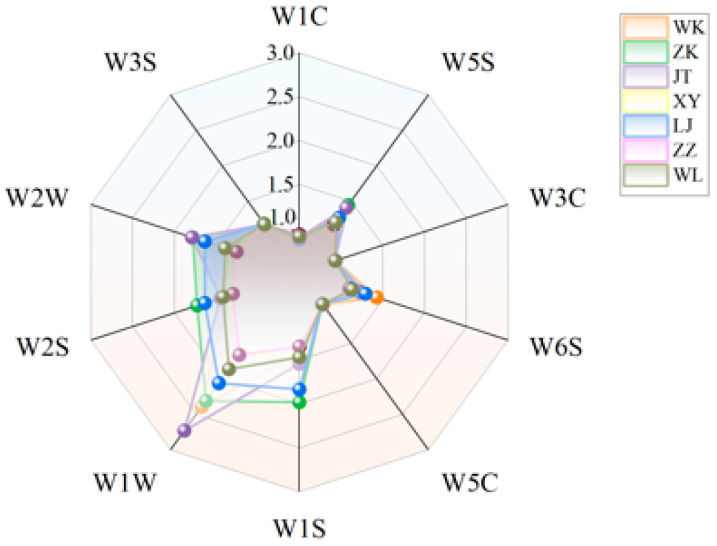
Radar plot of electronic nose sensor responses for cooked glutinous rice from seven cultivars. Sensor codes and their corresponding compound classes are listed in Appendix A (Table A3).

Electronic nose (E-nose) analysis was performed to characterize the volatile profiles of cooked glutinous rice across cultivars, with results visualized as radar plots (Figure 2). While responses from sensors W1C, W5S, W3C, W6S, W5C, and W3S showed no significant varietal differences, elevated signals from W1W (sulfur compounds), W2W (alcohols), and W1S (aldehydes/ketones) indicated that key volatiles primarily originated from sulfides, alcohols, and aldehyde/ketone derivatives. WK and JT exhibited the highest W1W and W2W responses, suggesting enriched sulfur-containing and alcoholic volatiles. Although the E-nose effectively captured global aroma profiles, it lacked compound-specific resolution, necessitating further SPME-GC-MS analysis.

##### Volatile Compound Profiling

In order to study the aroma characteristics of glutinous rice made from different varieties of glutinous rice, the SPME-GC-MS technique combined with multivariate statistical analysis was used to identify and analyze the volatile components of glutinous rice. The seven kinds of glutinous rice contained a total of 64 volatile compounds (Table A2), covering six major groups of alcohols, aldehydes, ketones, esters, alkanes, and heterocyclic compounds. Among them, aldehydes (24 species) dominated in both species and total amount, especially C6–C12 straight-chain saturated aldehydes (e.g., nonanal, octanal) and α,β-unsaturated aldehydes (e.g., (E)-2-octenal, (Z)-2-nonenal), whose low aroma threshold characteristics (generally lower than 0.01 mg/kg) determined the intensity and direction of the overall aroma of the glutinous rice; ketones (14 species) and alcohols (9 species) as the subclasses of volatiles; and ketone (14 species) and alkanol (9 species) as the subclasses of alcohols. Ketones (14 species) and alcohols (9 species) were secondary components, contributing to fruity, sweet and earthy aromas; esters and alkanes contributed weakly to the overall aroma; and heterocyclic compounds (e.g., 2-acetyl-1-pyrrolinoline, indole), although few, were key to defining the specificity of the varieties due to their very low thresholds.

A total of 17 compounds were characterized as key aroma-active compounds in glutinous rice based on their odor activity values (OAVs, the ratio of concentration to odor threshold), which were calculated to be greater than 1.0 (Table 8). Their distribution differences significantly drove the flavor variation between rice varieties. WL variety: Demonstrated overall dominance, synergistically enriching aldehydes (nonanal, unsaturated allyl aldehydes), alcohols (hexanol, 1-octen-3-ol), and heterocyclics (2-acetyl-1-pyrroline, 2-pentylfuran). This formed a high-intensity composite aroma profile featuring grassy, fruity, mushroom, and popcorn notes. Critically, the prominent expression of 2-acetyl-1-pyrroline established its high-quality flavored rice attribute [35]. ZZ variety: Excelled in aldehyde specificity. The co-expression of (E)-2-octenal (grassy) and (E)-2-dodecenal (citrus) constructed a refreshing grassy aroma system. This was supplemented by 2-pentylfuran [36]. The XY variety was significantly differentiated by the exclusive high content of indole (floral substance), which combines with (E, E)-2,4-decadienal (cucumber) to form a unique “floral-cucumber” aroma; the WK variety had a peak in the popcorn aroma marker (2-acetyl-1-pyrrolidine) but the aldehyde support was insufficient to create a single aroma [37]; the JT and LJ varieties were weak in overall aroma intensity due to the low content of key aldehydes, and the overall aroma intensity was weak in the form of a single aroma, with the aldehyde support being insufficient. JT and LJ, due to the low content of key aldehydes, had a weak overall aroma intensity, with fruity-earthy aromas dominated by alcohols (e.g., 1-octen-3-ol); and ZK was characterized by a transitional state, with medium-to-high levels of (E)-2-octenal to maintain the base green aroma.

**Figure 3:**
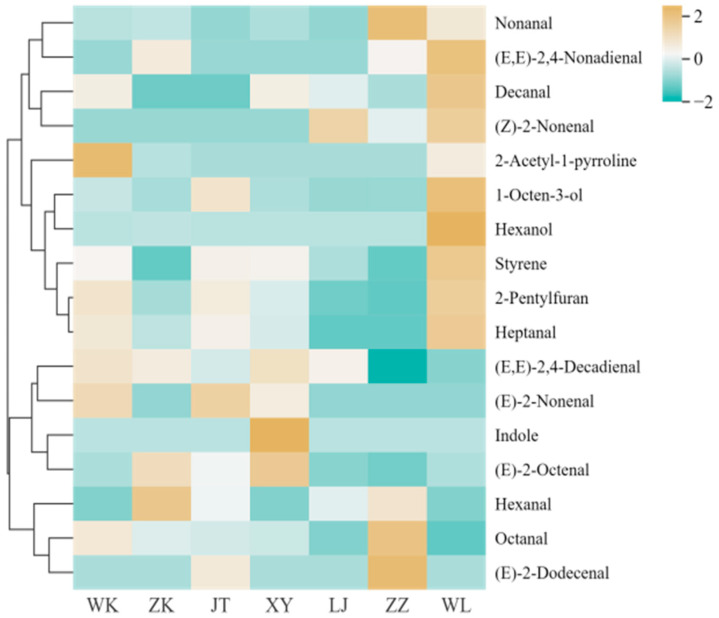
Percentage distribution of volatile compound content of different glutinous rice varieties. Data were based on GC-MS analysis (Table A2).

**Figure 4:**
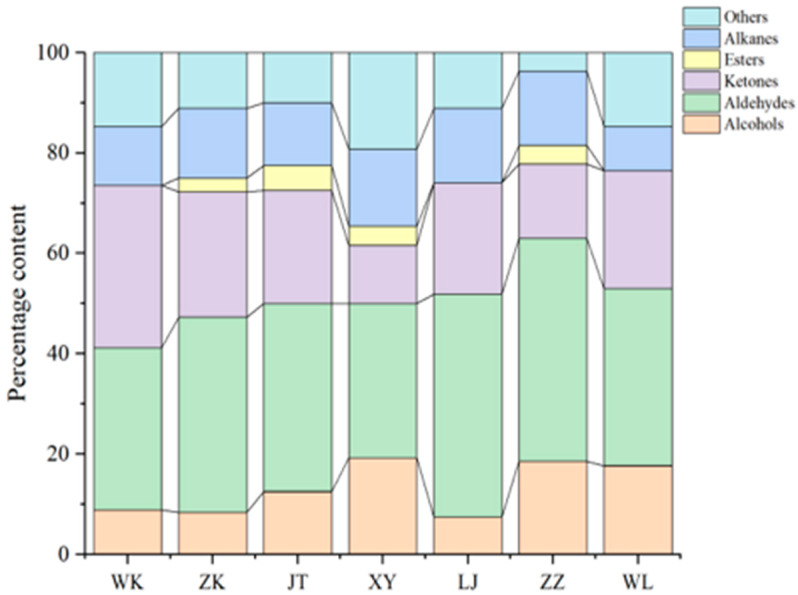
Heat map and clustering diagram of changes in the relative content of volatile substances obtained from different glutinous rice varieties with OAV > 1.

The percentage distribution of volatiles in Figure 3 verified the above chemical differentiation: WL and ZZ had a significantly higher proportion of aldehydes than the other varieties (more than 50% of the total), which was consistent with their high aroma activity; XY formed a “heterocyclic-dominant” special case due to the prominent proportion of indole (nearly 1/3 of the total); and the relative proportion of alcohols in JT/LJ was elevated, reflecting the tendency of their aroma simplification. The heatmap and hierarchical clustering in Figure 4 further revealed the association pattern of “chemical composition-aromatic type”: WL and ZZ were clustered into a “complex green aroma” due to the shared high expression of aldehydes (nonanal, (E)-2-octenal) and heterocyclic compounds (2-pentylfuran); JT and LJ were clustered into a “light fruity aroma” due to the low expression of aldehydes and medium alcohols; XY was clustered into a “floral-specific” due to the indole uniqueness; and WK and ZK were clustered into a “floral specific” due to the low expression of aldehydes and medium alcohols; and WK and ZK were clustered into a “floral specific” due to the indole uniqueness. WK and ZK were in a transition state due to the local dominance of 2-acetyl-1-pyrroline or a single aldehyde.

#### 3.3.4. Sensory Evaluation

The results of the sensory evaluation are shown in Table 9, and there were significant differences (*p* < 0.05) in the overall quality of the seven glutinous rice varieties. The WK cultivar demonstrated superior performance in total score and all key sensory attributes (aroma, appearance, palatability, taste, and cold rice texture), with its exceptional palatability and stable cold rice texture indicating advantages in flavor intensity and textural tolerance [38]. ZK ranked second in total score but exhibited markedly lower palatability and cold rice texture scores than WK, reflecting textural limitations. JT and LJ showed intermediate total scores, constrained by low aroma/appearance ratings (JT) and deficits in cold rice texture/taste (LJ). XY and ZZ scored the lowest, with significant deficiencies in core metrics (aroma, appearance, taste), particularly undermined by poor appearance and palatability. However, WL matched ZK in cold rice texture; its inferior aroma and appearance restricted the overall sensory performance.

In summary, WK exhibited outstanding potential as a premium glutinous rice cultivar due to its balanced sensory profile and high stability across critical parameters. Other cultivars require targeted improvements addressing specific defects (e.g., insufficient aroma, crumbly cold rice texture, or incohesive morphology) to enhance their properties.

## 4. Discussion

When integrating the multi-dimensional data, several consistent patterns emerged across varieties. Varieties with higher bound water content (WK and JT) exhibited greater hardness and received higher palatability scores, suggesting that water-binding capacity is a key determinant of textural quality. This observation aligns with previous findings that bound water contributes to starch granule stability and gel network formation during cooking [20,21]. Recent studies further confirmed that water mobility and distribution closely determine the texture stability and eating quality of glutinous rice [32]. Varieties with elevated free water (ZK and WL) showed a tendency toward lower overall sensory acceptance. High free water content may increase water activity and accelerate sensory deterioration, particularly during post-cooking storage [3]. From a flavor perspective, varieties with higher aldehyde content, particularly ZZ, consistently achieved higher sensory odor scores. Aldehydes such as nonanal and (E)-2-octenal are known to impart grassy and citrus-like notes, and their low odor thresholds make them critical contributors to rice aroma [35]. This is consistent with recent reviews indicating that aldehydes and key heterocyclic compounds dominate the characteristic aroma of glutinous rice products [39].

The thermal properties of the cultivars further support their processing suitability. WL exhibited the highest gelatinization temperatures (T_o_, T_p_, T_c_) and enthalpy (ΔH), suggesting higher structural order within its starch granules, which requires greater energy input for gelatinization [27]. This property makes WL suitable for high-temperature industrial processes such as extrusion or retort sterilization. In contrast, ZK and JT demonstrated lower onset and peak temperatures, indicative of rapid gelatinization kinetics, which is advantageous for instant food applications where quick rehydration is required [25]. Low gelatinization temperature and low retrogradation tendency have been recommended as key criteria for instant and ready-to-eat glutinous rice products [40]. The anti-retrogradation property of ZK, reflected in its low setback viscosity, further supports its use in frozen or ready-to-eat products [29].

Protein composition also played a significant role in varietal functionality. Differential protein fractions have been reported to significantly affect water-binding capacity and textural formation in rice products [41]. WK, with the highest total protein content and relatively high globulin fraction, exhibited superior water-binding capacity and textural stability. Globulins are known to enhance emulsifying and hydration properties, which may contribute to the compact texture observed in cooked WK rice [23]. Conversely, ZK contained a higher proportion of glutelin and prolamin, which are more hydrophobic and thermally stable, potentially contributing to its structural integrity during processing [24].

Comparisons with previous studies revealed both consistency and novelty. While varietal differences in pasting and thermal properties align with earlier reports on rice starch functionality [6,15], the present study extends these findings by linking them to water mobility (LF-NMR), protein fractionation (Osborne method), and comprehensive sensory and volatile profiling. This integrated approach provides a holistic understanding of variety-specific quality formation, offering a scientific basis for precision breeding and cultivar-specific industrial applications.

## 5. Conclusions

This study reveals the differentiated characteristics of seven glutinous rice varieties through multidimensional quality analysis. The integrated findings provide a basis for precision variety selection: WK, with its high protein content and excellent water-binding capacity, is recommended for traditional steamed products like zongzi and rice cakes, where textural stability is paramount. ZK, characterized by rapid gelatinization and anti-retrogradation properties, is highly suitable for instant food processing, such as ready-to-eat tangyuan. WL, despite its high peak viscosity and thermal stability, requires careful moisture control during processing due to its higher free water content. ZZ’s superior volatile profile makes it ideal for flavor-oriented products. This work establishes a framework for optimizing processing adaptability based on varietal characteristics.

## Figures and Tables

**Table 1:** Percentage of water in different states of glutinous rice.

Sample	*A*_21_/%	*A*_22_/%	*A*_23_/%
WK	3.08 ± 0.21 ^c^	95.18 ± 4.82 ^a^	1.74 ± 0.12 ^ab^
ZK	3.40 ± 0.15 ^bc^	94.65 ± 5.39 ^a^	1.95 ± 0.16 ^a^
JT	6.08 ± 0.61 ^a^	92.38 ± 4.94 ^a^	1.54 ± 0.07 ^abc^
XY	3.45 ± 0.27 ^bc^	95.07 ± 3.69 ^a^	1.47 ± 0.18 ^bc^
LJ	3.40 ± 0.38 ^d^	95.36 ± 3.31 ^a^	1.24 ± 0.08 ^c^
ZZ	5.25 ± 0.31 ^ab^	93.29 ± 4.12 ^a^	1.46 ± 0.08 ^bc^
WL	3.77 ± 0.13 ^bc^	94.41 ± 2.06 ^a^	1.82 ± 0.15 ^ab^

The results are expressed as the mean ± standard error (*n* = 3), and different lowercase letters in the same column indicate significant differences among the different glutinous rice varieties (*p* < 0.05).

**Table 2:** Protein content and percentage composition of graded extracted glutinous proteins.

Sample	Proteins/%	Albumin/%	Globulin/%	Prolamin/%	Glutenin/%
WK	8.37 ± 0.06 ^a^	8.02 ± 0.63 ^f^	11.51 ± 0.62 ^c^	6.25 ± 0.39 ^a^	49.73 ± 0.09 ^c^
ZK	7.98 ± 0.23 ^b^	13.3 ± 0.23 ^c^	12.07 ± 0.31 ^bc^	5.85 ± 0.42 ^a^	55.73 ± 1.31 ^a^
JT	7.75 ± 0.34 ^bc^	16.98 ± 0.61 ^a^	13.76 ± 0.26 ^a^	6.34 ± 0.33 ^a^	49.61 ± 1.21 ^c^
XY	7.44 ± 0.23 ^c^	11.47 ± 0.37 ^d^	12.66 ± 0.27 ^b^	1.49 ± 0.17 ^b^	42.15 ± 0.15 ^d^
LJ	7.08 ± 0.05 ^d^	9.67 ± 0.55 ^e^	8.86 ± 0.27 ^d^	1.52 ± 0.18 ^b^	54.22 ± 0.26 ^a^
ZZ	6.46 ± 0.14 ^e^	7.15 ± 0.52 ^f^	8.17 ± 0.09 ^e^	5.95 ± 0.33 ^a^	38.01 ± 0.57 ^e^
WL	6.51 ± 0.03 ^e^	14.93 ± 0.98 ^b^	11.43 ± 0.78 ^c^	6.34 ± 0.07 ^a^	52.59 ± 1.25 ^b^

The results are expressed as the mean ± standard error (*n* = 6), and different lowercase letters in the same column indicate significant differences among the different glutinous rice varieties (*p* < 0.05).

**Table 3:** Gel consistency and alkali elimination values of different glutinous rice cultivars.

Sample	Gel Consistency (mm)	Alkali Elimination Value (Grade)
WK	108.0 ± 2.0 ^c^	5.4 ± 0.3 ^c^
ZK	116.0 ± 0.0 ^a^	6.6 ± 0.2 ^a^
JT	102.0 ± 3.0 ^d^	6.2 ± 0.0 ^b^
XY	116.0 ± 1.0 ^a^	2.8 ± 0.1 ^f^
LJ	110.0 ± 3.0 ^b^	5.1 ± 0.2 ^c^
ZZ	100.0 ± 3.0 ^d^	3.6 ± 0.0 ^e^
WL	88.0 ± 1.0 ^e^	4.2 ± 0.2 ^d^

The results are expressed as the mean ± standard error (*n* = 6), and different lowercase letters in the same column indicate significant differences among the different glutinous rice varieties (*p* < 0.05).

**Table 4:** Thermal properties of different glutinous rice varieties.

Sample	T_o_ (°C)	T_p_ (°C)	T_c_ (°C)	ΔH (J/g)
WK	65.31 ± 0.21 ^c^	70.41 ± 0.01 ^c^	77.23 ± 0.72 ^bc^	9.32 ± 0.60 ^c^
ZK	62.08 ± 0.04 ^d^	68.10 ± 0.01 ^d^	73.72 ± 1.07 ^d^	10.58 ± 0.06 ^bc^
JT	62.68 ± 0.12 ^d^	67.41 ± 0.06 ^d^	72.82 ± 0.73 ^d^	8.72 ± 0.22 ^c^
XY	67.83 ± 0.76 ^b^	73.2 ± 0.52 ^b^	78.46 ± 0.02 ^b^	9.68 ± 0.40 ^c^
LJ	63.21 ± 1.63 ^d^	69.70 ± 1.03 ^c^	76.79 ± 0.96 ^c^	9.62 ± 1.51 ^c^
ZZ	65.66 ± 0.04 ^c^	72.36 ± 0.59 ^b^	78.58 ± 0.20 ^b^	12.38 ± 0.74 ^ab^
WL	69.62 ± 0.28 ^a^	76.55 ± 0.09 ^a^	84.17 ± 0.22 ^a^	14.15 ± 1.38 ^a^

The results are expressed as the mean ± standard error (*n* = 3), and different lowercase letters in the same column indicate significant differences among the different glutinous rice varieties (*p* < 0.05). T_o_ = Onset temperature; T_p_ = Peak temperature; T_c_ = Conclusion temperature; ΔH = Enthalpy change.

**Table 5:** Pasting properties of glutinous rice flour.

Sample	PV (mP·s)	MV (mP·s)	BV (mP·s)	FV (mP·s)	SV (mP·s)	GT (°C)
WK	933.00 ± 8.00 ^d^	154.00 ± 12.76 ^d^	784.00 ± 6.85 ^c^	537.00 ± 6.93 ^d^	384.00 ± 17.53 ^bc^	72.05 ± 0.36 ^c^
ZK	812.00 ± 11.00 ^e^	149.00 ± 7.03 ^d^	663.00 ± 12.68 ^d^	509.00 ± 36.00 ^d^	356.00 ± 37.56 ^c^	69.66 ± 0.42 ^e^
JT	856.00 ± 6.74 ^de^	85.00 ± 11.00 ^d^	776.00 ± 19.84 ^c^	562.00 ± 49.96 ^d^	477.00 ± 40.16 ^abc^	69.50 ± 0.37 ^e^
XY	1507.00 ± 63.28 ^c^	861.00 ± 71.79 ^c^	646.00 ± 25.31 ^d^	1426.00 ±93.21 ^c^	566.00 ± 92.97 ^a^	74.36 ± 0.58 ^b^
LJ	873.00 ± 32.00 ^de^	97.00 ± 72.28 ^d^	780.00 ± 48.19 ^c^	594.00 ± 48.00 ^d^	497.00 ± 112.28 ^ab^	70.97 ± 0.52 ^d^
ZZ	2194.00 ± 101.72 ^b^	1006.00 ± 32.15 ^b^	1188.00 ± 73.55 ^b^	1531.00 ± 30.20 ^b^	525.00 ± 46.69 ^a^	73.86 ± 0.54 ^b^
WL	2521.00 ± 65.78 ^a^	1172.00 ± 47.28 ^a^	1349.00 ± 13.62 ^a^	1745.00 ± 52.31 ^a^	574.00 ± 62.18 ^a^	76.27 ± 0.61 ^a^

The results are expressed as the mean ± standard error (*n* = 3), and different lowercase letters in the same column indicate significant differences among the different glutinous rice varieties (*p* < 0.05). PV = Peak viscosity; MV = Minimum viscosity; BV = Breakdown viscosity; FV = Final viscosity; SV = Setback viscosity; GT = Gelatinization temperature.

**Table 6:** Textural properties of steamed glutinous rice.

Sample	Hardness (g)	Springiness	Chewiness (mJ)	Gumminess (N)	Cohesiveness	Resilience
WK	74.88 ± 5.38 ^a^	0.59 ± 0.02 ^b^	31.48 ± 3.72 ^b^	46.90 ± 3.76 ^a^	0.57 ± 0.01 ^a^	0.28 ± 0.02 ^b^
ZK	78.66 ± 3.27 ^a^	0.58 ± 0.03 ^a^	32.10 ± 3.05 ^a^	49.78 ± 4.32 ^a^	0.59 ± 0.03 ^a^	0.29 ± 0.03 ^ab^
JT	56.79 ± 3.16 ^c^	0.50 ± 0.05 ^b^	17.50 ± 2.95 ^c^	30.20 ± 3.47 ^bc^	0.50 ± 0.01 ^c^	0.24 ± 0.01 ^bc^
XY	55.30 ± 4.93 ^c^	0.48 ± 0.02 ^b^	16.04 ± 3.64 ^c^	28.12 ± 2.08 ^c^	0.49 ± 0.02 ^c^	0.21 ± 0.01 ^c^
LJ	64.66 ± 2.06 ^b^	0.52 ± 0.04 ^b^	21.43 ± 2.86 ^bc^	35.42 ± 3.93 ^b^	0.54 ± 0.03 ^b^	0.22 ± 0.02 ^c^
ZZ	59.52 ± 3.14 ^c^	0.51 ± 0.03 ^b^	19.96 ± 2.62 ^c^	33.54 ± 2.46 ^bc^	0.55 ± 0.02 ^b^	0.25 ± 0.01 ^b^
WL	58.63 ± 3.09 ^c^	0.52 ± 0.03 ^ab^	16.91 ± 1.79 ^c^	29.32 ± 2.04 ^c^	0.53 ± 0.01 ^b^	0.28 ± 0.02 ^a^

The results are expressed as the mean ± standard error (*n* = 3), and different lowercase letters in the same column indicate significant differences among the different glutinous rice varieties (*p* < 0.05).

**Table 7:** Color parameters of steamed glutinous rice.

Sample	L*	a*	b*	W
WK	70.99 ± 2.37 ^a^	−0.41 ± 0.08 ^a^	8.43 ± 0.31 ^c^	69.79 ± 1.84 ^a^
ZK	68.95 ± 1.31 ^a^	−0.41 ± 0.06 ^a^	6.73 ± 0.27 ^d^	68.23 ± 2.25 ^a^
JT	57.05 ± 2.05 ^c^	−1.43 ± 0.01 ^b^	8.43 ± 0.37 ^c^	56.21 ± 2.94 ^c^
XY	65.21 ± 2.16 ^ab^	−1.43 ± 0.02 ^b^	9.79 ± 0.08 ^b^	63.83 ± 1.95 ^b^
LJ	60.24 ± 2.28 ^ab^	−1.27 ± 0.12 ^b^	9.74 ± 0.93 ^b^	59.04 ± 1.73 ^b^
ZZ	62.15 ± 2.07 ^b^	−0.79 ± 0.01 ^ab^	12.85 ± 0.42 ^a^	60.02 ± 1.98 ^b^
WL	56.71 ± 0.64 ^bc^	−1.77 ± 0.13 ^b^	10.81 ± 0.78 ^b^	55.35 ± 2.38 ^c^

The results are expressed as the mean ± standard error (*n* = 6), and different lowercase letters in the same column indicate significant differences among the different glutinous rice varieties (*p* < 0.05).

**Table 8:** The volatile aroma compounds with OAV > 1.0 in glutinous rice.

No.	Compounds	Odor Threshold (mg/kg)	Contents (μg/kg)						
			WK	ZK	JT	XY	LJ	ZZ	WL
1	Hexanol	0.0056	—	0.41 ± 0.08 ^b^	—	—	—	—	21.08 ± 11.28 ^a^
2	1-Octen-3-ol	0.0015	12.97 ± 1.65 ^c^	7.26 ± 0.28 ^a^	34.4 ± 16.47 ^bc^	8.53 ± 0.87 ^de^	4.26 ± 1.31 ^e^	4.44 ± 0.57 ^e^	56.48 ± 96.27 ^b^
3	Hexanal	0.005	—	34.71 ± 14.56 ^a^	14.58 ± 9.81 ^c^	—	12.64 ± 4.25 ^c^	23.02 ± 3.36 ^b^	—
4	Heptanal	0.0028	3.79 ± 0.99 ^b^	1.61 ± 0.32 ^d^	3.21 ± 0.45 ^b^	2.15 ± 0.29 ^c^	—	—	5.59 ± 3.4 ^a^
5	Octanal	0.000587	10.00 ± 2.19 ^a^	7.31 ± 0.49 ^b^	6.95 ± 0.88 ^bc^	6.69 ± 0.32 ^bc^	4.18 ± 1.47 ^d^	14.06 ± 3.84 ^a^	3.18 ± 1.77 ^d^
6	Nonanal	0.0058	89.06 ± 1.47 ^c^	99.22 ± 7.07 ^c^	54.83 ± 10.26 ^e^	79.82 ± 4.56 ^d^	53.34 ± 16.58 ^e^	338.74 ± 13.19 ^a^	206.77 ± 47.14 ^b^
7	(Z)-2-Nonenal	0.00002	—	—	—	—	22.79 ± 8.15 ^b^	8.51 ± 5.77 ^c^	24.49 ± 19.2 ^a^
8	(E)-2-Octenal	0.003	38.15 ± 5.29 ^d^	151.49 ± 52.31 ^b^	89.91 ± 56.72 ^c^	192.28 ± 31.11 ^a^	13.52 ± 4.84 ^e^	—	40.96 ± 29.46 ^d^
9	Decanal	0.003	46.06 ± 22.06 ^b^	—	—	45.32 ± 7.23 ^b^	31.41 ± 9.64 ^bc^	16.32 ± 0.18 ^d^	79.47 ± 42.38 ^a^
10	(E)-2-Nonenal	0.00019	16.23 ± 12.29 ^a^	—	18.11 ± 1.45 ^a^	11.22 ± 7.57 ^b^	—	—	—
11	(E,E)-2,4-Nonadienal	0.0001	—	3.33 ± 0.03 ^b^	—	—	—	2.65 ± 0.11 ^c^	6.58 ± 3.05 ^a^
12	(E)-2-Dodecenal	0.0014	—	—	2.13 ± 0.42 ^b^	—	—	4.72 ± 1.45 ^a^	—
13	(E,E)-2,4-Decadienal	0.000027	20.11 ± 2.63 ^a^	17.91 ± 0.26 ^b^	12.69 ± 3.91 ^c^	20.67 ± 0.39 ^a^	16.69 ± 2.62 ^b^	—	6.97 ± 3.48 ^d^
14	2-Pentylfuran	0.0058	63.65 ± 9.14 ^b^	29.35 ± 4.37 ^d^	58.26 ± 13.88 ^bc^	41.86 ± 2.92 ^c^	17.65 ± 5.88 ^e^	14.26 ± 2.1 ^e^	80.67 ± 42.34 ^a^
15	Styrene	0.0036	0.73 ± 0.09 ^b^	—	0.79 ± 0.1 ^b^	0.76 ± 0.27 ^b^	0.32 ± 0.09 ^c^	—	1.42 ± 0.61 ^a^
16	2-Acetyl-1-pyrroline	0.053	39.21 ± 29.1 ^a^	1.96 ± 0.07 ^c^	—	—	—	—	16.78 ± 11.42 ^b^
17	Indole	0.011	—	—	—	88.17 ± 13.67 ^a^	—	—	—

The results are expressed as the mean ± standard error (*n* = 3). Different lowercase letters in the same row indicate significant differences among cultivars (*p* < 0.05) as determined by one-way ANOVA with Duncan’s multiple comparison test. “—” indicates not detected or below the limit of quantification.

**Table 9:** Sensory evaluation of edible quality of steamed glutinous rice.

Sample	Odor (20 Points)	Morphology (20 Points)	Palatability (30 Points)	Taste (25 Points)	Cold Rice Texture (5 Points)	Total Score (100 Points)
WK	18.83 ± 0.75 ^a^	19.00 ± 0.63 ^a^	26.00 ± 1.41 ^a^	24.00 ± 0.63 ^a^	5.00 ± 0.00 ^a^	92.83 ± 2.86 ^a^
ZK	18.00 ± 0.63 ^a^	18.67 ± 1.03 ^a^	24.00 ± 0.63 ^b^	24.00 ± 0.63 ^a^	4.00 ± 0.89 ^b^	88.67 ± 2.66 ^b^
JT	16.83 ± 0.75 ^b^	17.00 ± 0.63 ^b^	24.00 ± 0.63 ^b^	22.83 ± 0.75 ^b^	4.00 ± 0.89 ^b^	84.67 ± 3.08 ^c^
XY	14.00 ± 0.63 ^d^	16.00 ± 0.63 ^c^	22.00 ± 0.63 ^d^	20.83 ± 0.75 ^d^	3.00 ± 0.63 ^c^	75.83 ± 2.93 ^f^
LJ	16.83 ± 0.75 ^b^	17.00 ± 0.63 ^b^	23.00 ± 0.63 ^c^	22.00 ± 0.63 ^c^	3.00 ± 0.63 ^c^	81.83 ± 2.64 ^cd^
ZZ	16.83 ± 0.75 ^b^	15.00 ± 0.63 ^d^	21.00 ± 0.63 ^e^	20.00 ± 0.63 ^e^	4.00 ± 0.63 ^b^	76.83 ± 2.93 ^ef^
WL	15.00 ± 0.63 ^c^	16.00 ± 0.63 ^c^	22.00 ± 0.63 ^d^	23.00 ± 0.89 ^b^	4.00 ± 0.63 ^b^	80.00 ± 2.61 ^de^

The results are expressed as the mean ± standard error (*n* = 10), and different lowercase letters in the same column indicate significant differences among the different glutinous rice varieties (*p* < 0.05).

## Data Availability

The original contributions presented in the study are included in the article, further inquiries can be directed to the corresponding author.

## Appendix A

**Table A1:** Scoring criteria and record sheet for sensory evaluation of glutinous rice.

Panelist ID:	Name:	Gender:	Age:	Place of Origin:	Date:	Time:
Primary Indicator & Score	Secondary Indicator & Score	Specific Characteristics & Scoring Criteria				Sample Score
Odor (20 Points)	Purity & Intensity (20 pts)	Has a characteristic rice aroma, rich and pronounced: 18–20 pts				
		Has a characteristic rice aroma, faintly fragrant: 15–17 pts				
		Has a characteristic rice aroma, very weak/indistinct: 12–14 pts				
		No distinct aroma, but no off-odors: 7–12 pts				
		Presence of off-odors: 0–6 pts				
Appearance (20 Points)	Color (7 pts)	Bright white: 6–7 pts				
		Normal/acceptable color: 4–5 pts				
		Yellowish or grayish: 0–3 pts				
	Glossiness (8 pts)	Shiny/glossy: 7–8 pts				
		Slightly glossy: 5–6 pts				
		Dull: 0–4 pts				
	Grain Integrity (5 pts)	Grains well-formed and intact, tight structure: 4–5 pts				
		Most grains intact, acceptable structure: 3 pts				
		Grains split or “exploded”: 0–2 pts				
Palatability (Texture) (30 Points)	Stickiness (10 pts)	Smooth, sticky but not adhesive to teeth: 8–10 pts				
		Sticky, slightly adhesive to teeth: 6–7 pts				
		Too sticky (adhesive) or not sticky enough: 0–5 pts				
	Elasticity (10 pts)	Chewy and elastic: 8–10 pts				
		Slightly elastic: 6–7 pts				
		Loose, hard, or gritty: 0–5 pts				
	Softness/Hardness (10 pts)	Optimal softness/hardness: 8–10 pts				
		Slightly too hard or too soft: 6–7 pts				
		Much too hard or too soft/mushy: 0–5 pts				
Taste (25 Points)	Purity & Persistence (25 pts)	Pronounced fragrant and sweet flavor during chewing: 22–25 pts				
		Faint fragrant and sweet flavor: 18–21 pts				
		No distinct fragrant or sweet flavor, but no off-flavors: 16–17 pts				
		No distinct flavor or presence of off-flavors: 0–15 pts				
Texture of Cold Cooked Rice (5 Points)	Cohesiveness, Elasticity & Hardness (5 pts)	Relatively loose, good elasticity, moderate hardness: 4–5 pts				
		Slightly cohesive, reduced elasticity, slightly harder: 2–3 pts				
		Compact/Congealed, poor elasticity, very hard: 0–1 pts				
TOTAL SCORE:						
Comments:						

**Table A2:** Volatile flavor content of glutinous rice.

Category	No.	CAS	Compounds	RIcal	Odor	Odor Threshold (mg/kg)	Identification
Alcohols	1	928-40-5	1,5-Hexanediol	1040	—	—	MS/RI
	2	71-41-0	Pentanol	1198	Sweet Aromatic	0.1502	MS/RI/O
	3	111-27-3	Hexanol	1296	Fruity, Green	0.0056	MS/RI/O
	4	589-35-5	3-Methyl-1-pentanol	1296	Fruity	0.0075	MS/RI/O
	5	36653-82-4	Hexadecanol	1364	—	—	MS/RI
	6	3913-2-8	2-Butyl-1-octanol	1375	—	—	MS/RI
	7	3391-86-4	1-Octen-3-ol	1391	Mushroom-like	0.0015	MS/RI/O
	8	4534-74-1	4-Ethylcyclohexanol	1430	—	—	MS/RI
	9	111-87-5	Octanol	1495	Citrusy, Sweet	0.1258	MS/RI/O
Aldehydes	10	66-25-1	Hexanal	1041	Green, Grassy	0.005	MS/RI/O
	11	111-71-7	Heptanal	1143	Fresh, Herbal	0.0028	MS/RI/O
	12	124-13-0	Octanal	1246	Citrusy, Herbal	0.000587	MS/RI/O
	13	57266-86-1	2-Heptenal	1274	—	—	MS/RI
	14	124-19-6	Nonanal	1350	Citrusy	0.0058	MS/RI/O
	15	123-15-9	2-Methylpental	1356	Fruity, Green	0.0016–0.0032	MS/RI/O
	16	2548-87-0	(E)-2-Octenal	1379	Fresh, Herbal	0.003	MS/RI/O
	17	112-54-9	Dodecanal	1407	Citrusy, Floral	0.01	MS/RI/O
	18	112-44-7	Undecanal	1408	Citrusy, Floral	0.0125	MS/RI/O
	19	4313-3-5	(E,E)-2,4-Heptadienal	1429	Green	—	MS/RI/O
	20	112-31-2	Decanal	1453	Citrusy, Floral	0.003	MS/RI/O
	21	100-52-7	Benzaldehyde	1453	Nutty	0.35	MS/RI/O
	22	60784-31-8	(Z)-2-Nonenal	1481	Cucumber-like	0.00002	MS/RI/O
	23	18829-56-6	(E)-2-Nonenal	1482	Cucumber-like	0.00019	MS/RI/O
	24	2497-25-8	(Z)-2-Decenal	1586	Fatty	—	MS/RI/O
	25	3913-81-3	(E)-2-Decenal	1588	Fatty	0.017–0.25	MS/RI/O
	26	13019-16-4	2-Butyl-2-octenal	1620	Fruity, Green	—	MS/RI/O
	27	5910-87-2	(E,E)-2,4-Nonadienal	1633	Fruity, Green	0.0001	MS/RI/O
	28	20407-84-5	(E)-2-Dodecenal	1692	Citrusy	0.0014	MS/RI/O
	29	2463-77-6	(E)-2-Undecenal	1694	Fruity	—	MS/RI/O
	30	2765-11-9	Pentadecanal	1763	—	—	MS/RI
	31	10486-19-8	Tridecanal	1765	Citrusy, Floral	0.07	MS/RI/O
	32	124-25-4	Tetradecanal	1975	Citrusy	0.11	MS/RI/O
	33	25152-84-5	(E,E)-2,4-Decadienal	1697	Cucumber-like	0.000027	MS/RI/O
Ketones	34	110-12-3	5-Methyl-2-hexanone	1138	—	—	MS/RI
	35	589-63-9	4-Octanone	1139	—	—	MS/RI
	36	105-42-0	4-Methyl-2-hexanone	1140	—	—	MS/RI
	37	111-13-7	2-Octanone	1241	Herbal	0.0502	MS/RI/O
	38	110-93-0	6-Methylhept-5-en-2-one	1286	Citrusy, Green	0.068	MS/RI/O
	39	98-86-2	Acetophenone	1329	Sweet Aromatic	0.065	MS/RI/O
	40	821-55-6	2-Nonanone	1344	Fruity, Sweet	0.041–0.082	MS/RI/O
	41	693-54-9	2-Decanone	1447	Citrusy	0.0083–0.041	MS/RI/O
	42	30086-02-3	(E,E)-3,5-octadien-2-one	1505	Fruity, Green	0.10–0.15	MS/RI/O
	43	6064-27-3	6-Dodecanone	1582	—	—	MS/RI
	44	6175-49-1	2-Dodecanone	1755	Citrusy	0.042–0.083	MS/RI/O
	45	593-08-8	2-Tridecanone	1752	Milky, Nutty	—	MS/RI/O
	46	3796-70-1	Geranylacetone	1790	Green, Woody	0.06	MS/RI/O
	47	2345-28-0	2-Pentadecanone	1963	Fatty	—	MS/RI/O
Esters	48	142-60-9	Propanoic acid, octylester	1250	Fruity, Sweet Aromatic	—	MS/RI/O
	49	142-91-6	Isopropyl palmitate	2189	—	—	MS/RI
	50	112-40-3	Dodecane	1176	—	—	MS/RI
	51	629-50-5	Tridecane	1279	—	—	MS/RI
	52	1502-38-1	Methylcyclooctane	1355	—	—	MS/RI
	53	629-59-4	Tetradecane	1477	—	—	MS/RI
	54	629-62-9	Pentadecane	1477	—	—	MS/RI
	55	544-76-3	Hexadecane	1581	—	—	MS/RI
	56	629-78-7	Heptadecane	1680	—	—	MS/RI
Others	57	4466-24-4	2-Butylfuran	1091	Fruity	0.005	MS/RI/O
	58	5989-27-5	(+)-Limonene	1158	Herbaceous	0.034	MS/RI/O
	59	3777-69-3	2-Pentylfuran	1190	Fruity, Green	0.0058	MS/RI/O
	60	100-42-5	Styrene	1205	Sweet Aromatic	0.0036	MS/RI/O
	61	85213-22-5	2-Acetyl-1-pyrroline	1281	Popcorn-like	0.000053	MS/RI/O
	62	4861-58-9	2-Pentylthiophene	1413	Hazelnut-like, Roasted Corn	—	MS/RI/O
	63	496-16-2	2,3-Dihydrobenzofuran	2253	—	—	MS/RI
	64	120-72-9	Indole	2307	Floral	0.011	MS/RI/O

MS = Mass spectrometry; RI = Retention index calculated based on GC analysis; O = Odor characteristics confirmed by olfactory analysis; CAS, Chemical Abstracts Service.

**Table A3:** Electronic nose (PEN3) sensor array and their corresponding compound classes.

Sensor Code	Description	Sensitive to
W1C	Aromatic	Benzene compounds
W5S	Broad-range	Nitrogen oxides
W3C	Aromatic	Ammonia, aromatic compounds
W6S	Hydrogen	Hydrogen hydrides
W5C	Arom-aliph	Short-chain alkanes, aromatic compounds
W1S	Broad-methane	Methyl compounds
W1W	Sulfur-organic	Inorganic sulfides
W2S	Broad-alcohol	Alcohols, aldehydes, ketones
W2W	Sulfur-chlorine	Organic sulfides, aromatic compounds
W3S	Methane-aliph	Long-chain alkanes
